# Sensitive, Efficient and Portable Analysis of Molecular Exchange Processes by Hyperpolarized Ultrafast NMR

**DOI:** 10.1002/anie.202203957

**Published:** 2022-05-17

**Authors:** Yashu Kharbanda, Mateusz Urbańczyk, Vladimir V. Zhivonitko, Sarah Mailhiot, Mikko I. Kettunen, Ville‐Veikko Telkki

**Affiliations:** ^1^ NMR Research Unit University of Oulu Oulu 90540 Finland; ^2^ Institute of Physical Chemistry Polish Academy of Sciences Warsaw Poland; ^3^ Kuopio Biomedical Imaging Unit A.I. Virtanen Institute for Molecular Sciences University of Eastern Finland Kuopio Finland

**Keywords:** Analytical Methods, Diffusion, Hyperpolarization, Molecular Exchange, Ultrafast NMR Spectroscopy

## Abstract

Molecular exchange processes are ubiquitous in nature. Here, we introduce a method to analyze exchange processes by using low‐cost, portable, single‐sided NMR instruments. The inherent magnetic field inhomogeneity of the single‐sided instruments is exploited to achieve diffusion contrast of exchange sites and spatial encoding of 2D data. This so‐called ultrafast diffusion exchange spectroscopy method shortens the experiment time by two to four orders of magnitude. Furthermore, because full 2D data are measured in a single scan (in a fraction of a second), the sensitivity of the experiment can be improved by several orders of magnitude using so‐called nuclear spin hyperpolarization methods (in this case, dissolution dynamic nuclear polarization). As the first demonstration of the feasibility of the method in various applications, we show that the method enables quantification of intra‐ and extracellular exchange of water in a yeast cell suspension.

## Graphical abstract

A hyperpolarized ultrafast diffusion exchange spectroscopy method is introduced, which allows highly sensitive and efficient quantification of molecular exchange rates by using low‐cost, low‐field, single‐sided and portable NMR instruments. The method can be used to investigate exchange processes in many fields ranging from intra‐extracellular exchange of metabolites to atmospheric surfactant solutions.

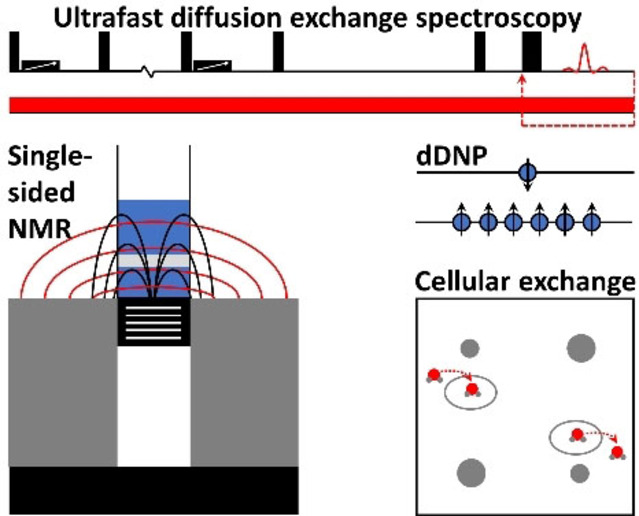

Exchange of molecules between different physical or chemical environments plays a crucial role in a plethora of fundamental natural processes ranging from chemical reactions and catalysis to breathing and metabolic processes. Molecular level observation and analysis of exchange processes is often challenging, as typically the processes take place inside an opaque solid matrix.

Nuclear magnetic resonance (NMR) spectroscopy[^1^, ^2^] allows versatile and non‐invasive characterization of chemical structures and dynamics of molecules even inside optically opaque materials, and 2D NMR has been broadly exploited in the investigation of molecular exchange.^[3]^ However, NMR also has some weaknesses that limit its use. For example, NMR is relatively insensitive due to small thermal polarization of nuclei; multidimensional experiments, which enable one to correlate various spectral parameters and study molecular exchange phenomena, are slow, because each data point of indirect dimension must be collected in repeated measurements; and high‐field NMR instruments are expensive, bulky, and not portable.

Sensitivity of NMR can be improved by several orders of magnitude by modern nuclear spin hyperpolarization techniques.[^4^, ^5^, ^6^, ^7^] Dissolution dynamic nuclear polarization (dDNP)^[4]^ is the most diverse polarization technique, as it allows the production of hyperpolarized liquid samples of many different nuclei. The technique has been broadly exploited in chemistry and biochemistry, for example, in determining metabolic pathways, as well as in vitro and in vivo imaging of metabolic fluxes.[^8^, ^9^]

Multidimensional experiments can be accelerated by spatial encoding of an indirect dimension into layers of a sample.[^10^, ^11^] This so‐called ultrafast (UF) NMR approach allows single‐scan measurement of 2D NMR data, reducing experiment time by one to three orders of magnitude.[^12^, ^13^, ^14^] Furthermore, it significantly facilitates the use of nuclear spin hyperpolarization, as the measurement and hyperpolarization process do not need to be repeated.^[15]^ For example, dDNP process takes typically from minutes to hours, preventing in practice conventional multidimensional experiments requiring tens to hundreds of repetitions. However, the single‐scan UF approach allows hyperpolarized 2D experiments. Although the spatial encoding lowers the sensitivity of a 2D experiment, because the data for a given evolution time is measured from a thin layer of sample, rather than the whole sample as in a conventional experiment, the sensitivity boost provided by hyperpolarization is much higher, leading to significant overall sensitivity enhancement.

Single‐sided, low‐field NMR instruments are attractive, as they are much cheaper than high‐field instruments and they are readily portable. Furthermore, they can be used for on‐site studies of surfaces of samples without size and geometry restrictions posed by the bore holes of the high‐field spectrometers.^[16]^ Typical probing depth varies from millimeters to a couple of centimeters. Single‐sided NMR has been exploited, e.g., in well‐logging^[17]^ and in the characterization of paintings,^[18]^ coatings,^[19]^ buildings,^[20]^ food,^[21]^ and human skin.^[22]^

The magnetic field of single‐sided instruments is inhomogeneous, preventing high‐resolution NMR spectroscopy, but allowing relaxation and diffusion NMR (so‐called Laplace NMR, L NMR) experiments, which rely on spin echoes. L NMR provides detailed information about molecular rotational and translational motion as well as local physical or chemical environment of nuclei.[^16^, ^23^] Multidimensional L NMR experiments enable correlation of different relaxation and diffusion parameters and study molecular exchange via relaxation or diffusion contrast even when the exchange pools are not resolved in spectrum.^[23]^ We have shown that multidimensional L NMR experiments can also be accelerated by spatial encoding, and this UF L NMR approach can be exploited in various applications ranging from porous material to cell metabolism and surfactant solutions relevant in aerosol research.[^24^, ^25^, ^26^, ^27^, ^28^, ^29^, ^30^, ^31^] Recently, we demonstrated that UF *T*_1_‐*T*_2_ (longitudinal and transverse relaxation) and *D*‐*T*_2_ (diffusion and transverse relaxation) correlation experiments are possible with a single‐sided magnet, when the inherent magnetic field homogeneity is exploited in spatial encoding.[^28^, ^29^] With hyperpolarization, even single‐scan 2D UF L NMR experiments became feasible, regardless of the low magnetic field of the single‐sided spectrometer.^[28]^

Here, we demonstrate for the first time that UF NMR exchange experiments are feasible with single‐sided magnets. Specifically, we show that it is possible to perform UF diffusion exchange spectroscopy (DEXSY) with a single‐sided magnet to investigate molecular exchange processes. Furthermore, we demonstrate that the huge sensitivity enhancement (four to five orders of magnitude) provided by dDNP allows single‐scan DEXSY experiments regardless of the low magnetic field of the single‐sided instrument. Therefore, the work provides novel analytical methodology for studying molecular exchange in various multidisciplinary applications, and it contributes to overcome simultaneously all the three major challenges of NMR listed above: slowness of multidimensional experiments, low sensitivity of NMR and restricted mobility of advanced NMR analysis.

The conventional DEXSY NMR experiment, which includes two diffusion‐encoding blocks separated by the mixing time *τ*_M_,^[32]^ is very time‐consuming, as the data of both indirect and direct dimensions are collected point‐by‐point. If the indirect and direct dimensions include *M* and *N* points, the measurement must be repeated by *M*×*N* times multiplied by the number of scans needed for accumulating signal and phase cycling. Typically, the number of points collected for each dimension is 10 to 100, requiring at least 100 to 10 000 repetitions and leading to very long experiment times.

Recently, we introduced a single‐scan, UF DEXSY experiment for high‐field instruments relying on spatial encoding of the indirect dimension and single‐scan detection.^[30]^ The spatial encoding block includes frequency‐swept π/2 and π pulses forming a double spin echo, accompanied with bipolar gradients, which make the double spin echo time linearly dependent on position and provide diffusion contrast. The single‐scan detection relies on a CPMG^[33]^ loop with gradient pulses, which, on the one hand, allow for reading the spatial encoding, and, on the other hand, provide diffusion contrast for the direct dimension.

The high‐field UF DEXSY experiment described above cannot be implemented for single‐sided NMR instruments having only a constant gradient, because the experiment requires bipolar pulsed gradients. In Figure 1a, we introduce an alternative pulse sequence for UF DEXSY experiments, which is compatible with single‐sided NMR instruments. In this version, the spatial encoding relies on stimulated echo, instead of double spin echo, similarly to the UF *D*‐*T*_2_ experiment for single‐sided instruments reported earlier.^[29]^ Due to the frequency‐swept chirp π refocusing pulse, the effective length of the gradient pulse becomes linearly dependent on position, being zero at the top‐most layer and maximum at the bottom‐most layer.^[31]^ Therefore, the longitudinal magnetization profile in the beginning of the mixing period *τ*_M_ is equivalent to the magnetization decay curve as a function of gradient strength in a conventional diffusion experiment. After the mixing period, the data are read in a single scan using the CPMG loop with a (constant) gradient like in the high‐field UF DEXSY experiment.

**Figure 1:**
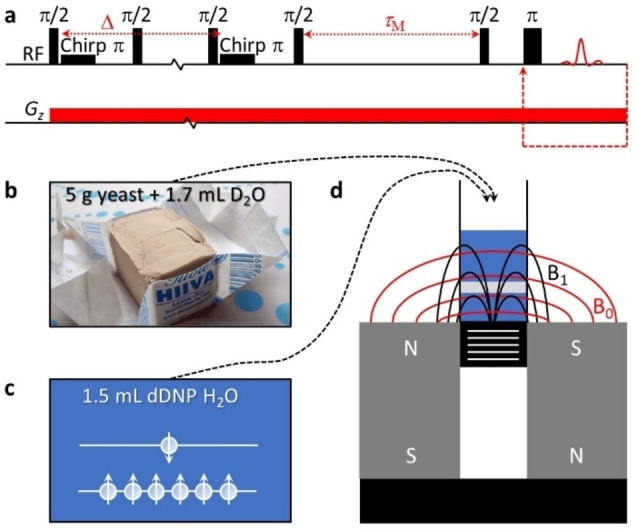
Ultrafast diffusion exchange spectroscopy (UF DEXSY) of water in a yeast cell suspension with a single‐sided NMR spectrometer. a) Pulse sequence for a constant gradient UF DEXSY experiment. b) A mixture of 5 g of fresh yeast and 1.7 mL of D_2_O was added to a sample vial with inner diameter of 2.5 cm. c) Thereafter, 1.5 mL of hyperpolarized water was added to the vial, and the UF DEXSY experiment was performed for 1–2 s after the addition. d) Illustration of the single‐sided NMR spectrometer and the sample vial on top of it. The gray slab inside the sample vial visualizes the sensitive region of the NMR coil. The magnetic field and ^1^H resonance frequency of the single‐sided spectrometer are 0.3 T and 13 MHz.

We exploited the sequence to study intra‐ and extracellular exchange of water in a yeast cell suspension. First, we mixed 5 g of fresh yeast with 1.7 mL of heavy water (D_2_O) in a vial to make it more fluid (Figure 1b). Then we placed the vial on the top of a single sided NMR instrument (Magritek NMR‐MOUSE PM25) and introduced 1.5 mL of dDNP hyperpolarized water into the vial (Figures 1c and d). The UF DEXSY experiment was performed 1–2 s after the introduction. To obtain more accurate information about the exchange process, the UF DEXSY experiment was repeated with three different mixing times, *τ*_M_=10, 30 and 100 ms. A new hyperpolarized sample was prepared before each repetition. The single‐scan UF DEXSY experiment took only 55–145 ms, depending on the mixing time.

The raw data of the UF DEXSY experiment with *τ*_M_=10 ms after the Fourier transform along the spatial encoding dimension is shown in Figure 2a. The figure was zoomed to cover only the 93 kHz region affected by the chirp pulse, corresponding to the 300 μm spatial encoding region. Signal‐to‐noise ratio (SNR) is relatively high (about 280), considering that the 2D data was measured in a single scan using a low‐field (0.3 T, 13 MHz) single‐sided instrument. The sensitivity enhancement provided by dDNP as compared to thermal polarization was estimated to be four to five orders of magnitude even after the signal decay during the short (1–2 s) delay between the injection of hyperpolarized H_2_O and measurement due to relaxation (*T*_1_ relaxation time was measured to be about 300 ms).

**Figure 2:**
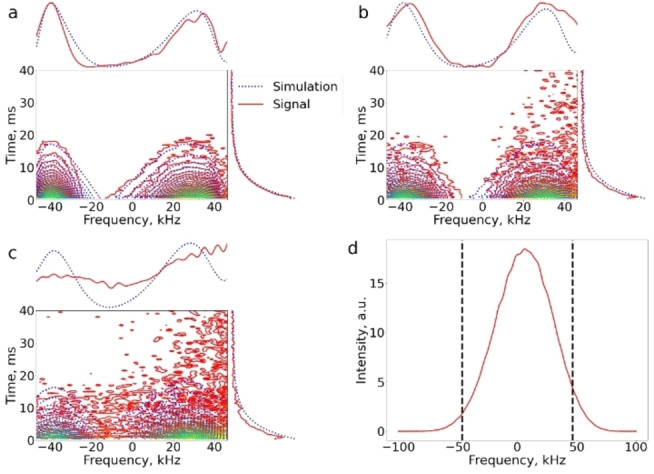
Raw data of UF DEXSY measurements of dDNP hyperpolarized water in a yeast cell suspension after a Fourier transformation along the spatial encoding direction. a) Mixing time *τ*_M_=10 ms. b) *τ*_M_=30 ms. c) *τ*_M_=100 ms. The first row and column of the data are plotted on the top and right, respectively. Red solid lines: experimental data (maximum signal in each experiment was normalized to 1). Blue dashed lines: simulated data. d) 1D spin‐echo MR image of a homogeneous water sample, representing the coil excitation detection sensitivity profile. Vertical black dashed lines indicate the bandwidth of the chirp pulses used in spatial encoding.

The spatially encoded diffusion decay curve, represented by Equation (38) in Ref. [31], is clearly visible in the first row shown on the top of Figure 2a. Note that in these experiments, the chirp refocusing pulse was swept from low to high frequencies, and therefore the spatially encoded diffusion curve decays from right to left. As can be seen from the first column plotted on the right of Figure 2a, the data in the vertical CPMG direction are decaying exponentially due to diffusion as shown by Equation (43) in Ref. [31]. Diffusion decay dominates over *T*_2_ decay in the CPMG loop due to very strong constant gradient (7.28 T m^−1^) of the single‐sided instrument.

A closer look at Figure 2a reveals that the diffusion decay curve in the spatial encoding direction is not perfectly ideal: on the right edge, the intensity is significantly lowered, although ideally it should correspond to the maximum intensity; on the left edge, the signal intensity is high, although ideally it should correspond to the minimum intensity. These deviations from the ideal behavior are even more pronounced in the UF DEXSY raw data of the longer mixing time experiments (Figures 2b and c).

The deviations are predominantly a consequence of the following three factors: 1. Due to very inhomogeneous *B*_1_ field of the single‐sided NMR instrument, both the excitation pulse flip angles and detection sensitivity are strongly dependent on position. 2. The frequency‐swept chirp pulses work non‐ideally in the beginning and at the end of sweeps, causing artefacts in the edges of the spatial encoding region. 3. The sensitive region of the single‐sided instrument (see Figure 1d) and the spatial encoding region (here 300 μm) are comparable with the displacements of the molecules due to diffusion during the mixing time *τ*_M_, and therefore there is a significant mixing of spatially encoded layers, as well as inflow of unencoded and outflow of encoded hyperpolarized molecules.

As explained in detail in the Supporting Information, it is possible to simulate all these effects. The excitation detection profile of the NMR coil was determined from an MR image of a homogeneous water sample (Figure 2d). Based on experimental observations, the effective *B*_1_ field of the chirp pulses was approximated to decrease close to zero around the beginning and end of the chirp, and the decreases were modelled by Gaussian distributions. The in‐ and outflow of molecules during the mixing time was modelled by diffusion propagators.^[23]^ In the data analysis, we assumed that the sample includes two diffusion components corresponding to intra‐ and extracellular water. This was validated by reference hyperpolarized CPMG diffusion measurements by the single‐sided instrument in identical conditions,^[30]^ which indicated that the intra‐ and extracellular diffusion coefficients are about 1.4 and 3.6×10^−9^ m^2^ s^−1^, respectively. The *D* values are high because of the elevated temperature of hyperpolarized water after dissolution (approximately 40 °C).^[34]^ In the UF DEXSY data simulations, *D* values were fixed to be equal to the reference measurements. This was done to improve the accuracy of the analysis, although, theoretically, if sensitivity and data quality is good enough, the method does not require prior knowledge about the sites. The simulated UF DEXSY data are plotted along with the experimental data in Figures 2a–c.

Figures 3a–c show 2D exchange maps resulting from the simulation of the experimental UF DEXSY data. The off‐diagonal cross‐peaks reveal that hyperpolarized water molecules exchange between the intra‐ and extracellular pools. The relative signal intensities of the diagonal and cross‐peaks as a function of mixing time are plotted in Figure 3d. The sum of the diagonal and cross‐peak signal intensities in each experiment was normalized to be one, as the intensities of the different experiments are not quantitatively comparable due to varying degree of hyperpolarization. The cross‐peak signal intensity increases with increasing mixing time, as more molecules change their pool due to the extended exchange time period. Figure 3d shows the fit of a two‐site exchange model^[3]^ with the experimental intensities. In the fit, *T*_1_ relaxation time was set to infinite, as overall *T*_1_ decay was effectively eliminated from the signal intensities in the normalization process. The fit resulted in the intra‐extracellular exchange rate of *k*=14±2 s^−1^ (*k*_intra_=3.4±1.0 s^−1^, *k*_extra_=11±2 s^−1^) and the relative population of the intracellular pool of 0.24±0.03. Therefore, the intracellular lifetime is *τ*_intra_=1/*k*_intra_=300±100 ms, which is in good agreement with the corresponding values reported in literature.^[35]^

**Figure 3:**
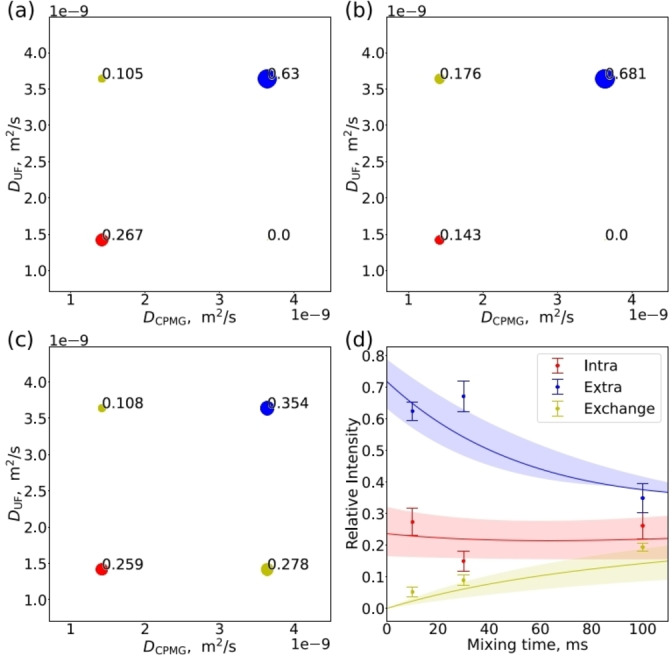
UF DEXSY maps measured with mixing time *τ*_M_ of a) 10, b) 30 and c) 100 ms. The diagonal peaks correspond to intra‐ and extracellular pools, while the off‐diagonal cross‐peaks reveal exchange between the two pools. The size of the circles as well as the numbers next to the circles represent relative intensities of the peaks. d) Relative peak intensities as a function of mixing time. Fits of the two‐site exchange model are shown by solid lines. Shadows represent errors of the fit.

In conclusion, we demonstrated the feasibility of the first ultrafast 2D exchange NMR experiment, UF DEXSY, with a single‐sided NMR instrument. Combination of the UF DEXSY method with the huge (four to five orders of magnitude) sensitivity enhancement provided by the dDNP method allowed a single scan 2D exchange measurement in a fraction of a second. The method was successfully exploited in the quantification of intra‐ and extracellular exchange of water in a yeast cell suspension. The methodology provides help for three major challenges of NMR analysis: low sensitivity, slowness, and non‐portability. Furthermore, it fosters advanced NMR analysis with low‐cost NMR instruments. We note that the dDNP method used in this work is neither affordable nor portable. However, PHIP, SABRE and SEOP techniques allow low‐cost, portable hyperpolarization.[^36^, ^37^, ^38^] Furthermore, researchers are developing techniques to extend the lifetime of hyperpolarization, allowing transport of DNP samples to different location for NMR observation.^[39]^ The UF DEXSY method offers promising prospect for in situ*/*in vivo studies of molecular exchange processes ubiquitous in various disciplines. Potential application fields include cellular metabolism,[^8^, ^9^, ^27^] fluid exchange processes in porous materials used in catalysis and selective adsorption,[^25^, ^28^, ^29^] aggregation of surfactants relevant in industry and aerosol research^[30]^ etc.

## Conflict of interest

The authors declare no conflict of interest.

## Supporting information

As a service to our authors and readers, this journal provides supporting information supplied by the authors. Such materials are peer reviewed and may be re‐organized for online delivery, but are not copy‐edited or typeset. Technical support issues arising from supporting information (other than missing files) should be addressed to the authors.

Supporting Information

## Data Availability

The Supporting Information includes description of experimental details and UF DEXSY data simulations. The datasets and analysis scripts used in this article are available in zenodo repository at: https://doi.org/10.5281/zenodo.5560815.
