# Supplementary material for: Sensitive, Efficient and Portable Analysis of Molecular Exchange Processes by Hyperpolarized Ultrafast NMR
Source: Angew Chem Int Ed Engl. 2022 May 17;61(28):e202203957. doi: 10.1002/anie.202203957 (PMC9400989; doi:10.1002/anie.202203957)
Supplement: Supplementary file 1 — Supporting Information [file ANIE-61-0-s001.pdf]

Angewandte Chemie
Eine Zeitschrift der Gesellschaft Deutscher Chemiker

Supporting Information

Sensitive, Efficient and Portable Analysis of Molecular Exchange Processes by Hyperpolarized Ultrafast NMR

Y. Kharbanda, M. Urbańczyk, V. V. Zhivonitko, S. Mailhiot, M. I. Kettunen, V.-V. Telkki**

Methods

Experimental

UF DEXSY NMR experiments. The UF DEXSY NMR experiments were performed with a low-field, single-sided NMR instrument Magritek PM25 profile NMR-MOUSE. The ^1H resonance frequency of the NMR-MOUSE is 13 MHz, the constant gradient strength is 7.28 T/m, and the maximum probing depth is 25 mm. The probing depth of 5 mm was used in the UF DEXSY experiments. The pulse sequence for the UF DEXSY experiment is shown in Figure 1. The length of the hard $\pi/2$ and π pulses was 4.5 μs (double power for the π pulses), the length of the frequency-swept chirp π refocusing pulse was 300 μs, the chirp bandwidth was 93 kHz, the time τ between the first two $\pi/2$ pulses was 600 μs, the diffusion delay Δ was 4 ms, the echo time and the number of echoes in the CPMG loop were 500 μs and 200, respectively, and the mixing time τ_M in three different experiments was 10, 30 and 100 ms. The UF DEXSY NMR experiments were performed for 1-2 s after insertion of hyperpolarized water into the yeast cell suspension. Experiment time was 55-145 ms, depending on the mixing time.

Yeast sample. Fresh baker's yeast, manufactured by Suomen Hiiva, was purchased from a local grocery store Prisma. According to the manufacturer, one gram of yeast includes about 10^9 yeast cells. The cell size is approximately 10 μm. The yeast was bought 0-3 days before the experiments and stored in a fridge at around 5°C. According to Parkkinen *et al.*,[1] the fraction of dead cells in a similar yeast is only 0-2% if the yeast is stored at 5°C less than 16 days. First, 5 g of the yeast was mixed with 1.7 mL of heavy water (D_2O) in a vial with inner diameter of 2.5 cm. We note that the added D_2O was not isotonic as it did not contain salt. However, the initial amount of (isotonic) water in the yeast sample was higher than the amount of added D_2O, and therefore it did not change osmotic pressure drastically. According to literature, yeast cells do not burst in a hypo-osmotic

environment.[2,3] Then the vial was placed on the top of the NMR-MOUSE, and 1.5 mL of dDNP hyperpolarized water was introduced into the vial before the UF DEXSY experiment.

Dissolution dynamic nuclear polarization. All reagents were purchased from Sigma-Aldrich (Darmstadt, Germany) unless otherwise mentioned. 200 µL aliquets consisting of MilliQ water and glycerol mix (3:2, v:v) and 25 mM 4-hydroxy-2,2,6,6-tetramethylpiperidin-1-oxyl (TEMPOL)[4] were hyperpolarized at 1.35 K, 6.7 T and 187.8 GHz for 1.5 h in a HYPERMAG polarizer (Technical University of Denmark, Copenhagen, Denmark).[5] The frozen sample was dissolved using 13 mL of 100mM sodium ascorbate containing 0.1 g/L ethylenediaminetetraacetic acid (EDTA) in D_2O (99.90%, Euriso-top, Saint-Aubin, France) to yield ~1.3 M final water concentration.

UF DEXSY simulations

Theoretical background for the UF DEXSY data simulations

The excitation-detection profile of NMR-MOUSE was estimated by measuring a 1D spin-echo image of a doped water sample, see Figure 2b. As illustrated in Figure S1, the profile can be approximated by a Gaussian function with mean of 6.3 kHz and standard deviation of 23.4 kHz. Note that the profile shown in Figure S1 is approximative, more precise profile (shown in Figure 2b) measured with imaging parameters identical to the UF DEXSY measurements were used in the analysis explained in the next section.

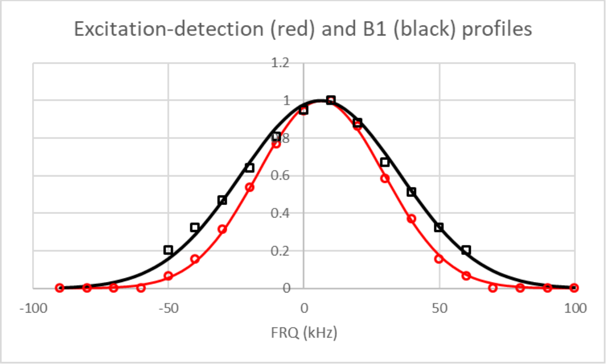

Figure S1. Excitation-detection (1D spin-echo image of a doped water sample, red circles) and B_1 (black squares) profiles of NMR-MOUSE. Solid lines: Gaussian fits.

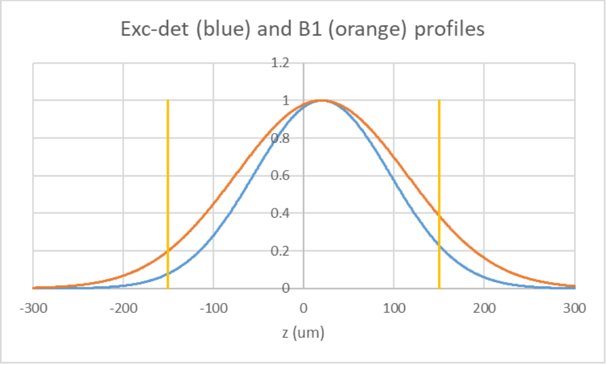

Figure S2. Excitation-detection (blue) and B_1 (orange) as a function of position. Vertical lines show the encoding region when the chirp bandwidth is 93 kHz. The width of the spatial encoding region is 300 μm.

Signal in the excitation-detection curve can be approximated by the following equation:

$$S(z) = \sin\left[\frac{\pi B_1(z)}{2}\right] \; B_1(z), \tag{1}$$

where B_1 is the relative B_1 field strength ($0 \leq B_1 \leq 1$, 1 corresponds to B_1 value of the $\pi/2$ excitation pulse). The sine term takes into account the position dependent excitation pulse angle, and the B_1 term accounts for the detection sensitivity, which is proportional to B_1 according to the principle of reciprocity.[6] Eq. 1 does not take into account the effect of selectivity of the hard π refocusing pulse.

The excitation-detection profile was converted to B_1 profile using Eq. 1 (see Figure S1). B_1 profile was approximated by a Gaussian function with mean of 6.1 kHz and width of 29.3 kHz (see Figures S1 and S2).

If we assume that the flip angle of $\pi/2$ excitation pulse is exactly 90°, when $B_1 = 1$, then the spatially dependent flip angle is

$$\theta(z) = \frac{\pi B_1(z)}{2}. \tag{2}$$

If the excitation pulse flip angle is less than 90°, then the transverse magnetization after the pulse is less than the original hyperpolarized magnetization M along the z-axis before the pulse, and some longitudinal magnetization remains along the z-axis, as illustrated in Figure S3. Only the transverse magnetization experiences the effect of the gradient pulse and becomes spatially encoded. As there are altogether four $\pi/2$ pulses in the spatial encoding part of the UF-DEXSY sequence, the encoded magnetization M_e stored along the z-axis for the mixing period is proportional to $\sin^4\theta(z)$, while the non-encoded z-magnetization M_{ne} is proportional to $\cos^4\theta(z)$ (see Figure S3).

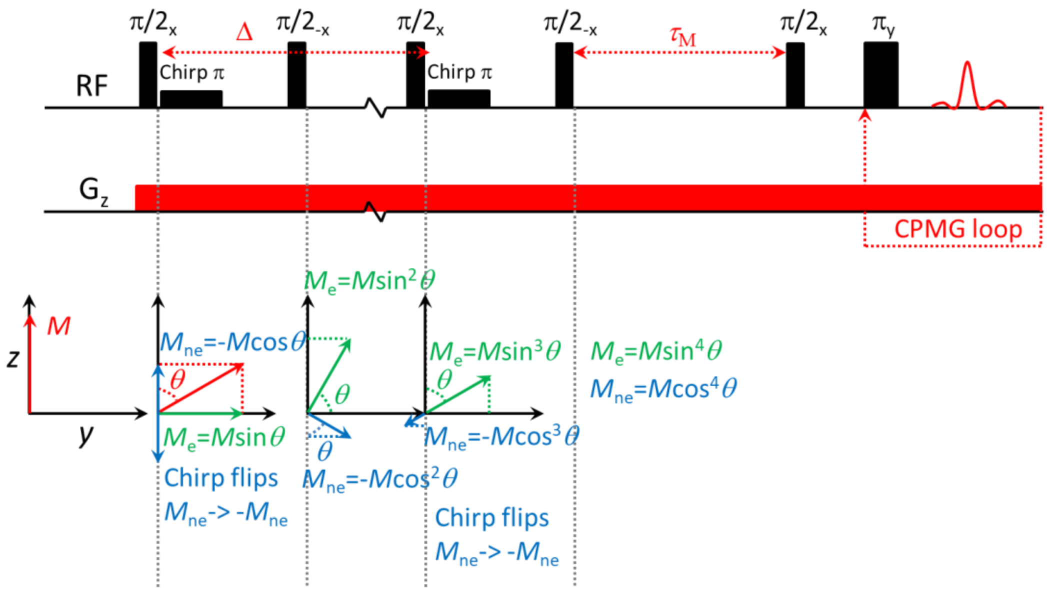

Figure S3. Pulse sequence for UF DEXSY and behavior of hyperpolarized magnetization ***M*** during the experiment. M_e and M_{ne} refer to encoded and non-encoded magnetization, respectively.

The spatially dependent weighting factors for encoded [$\sin^4\theta(z)$] and non-encoded [$\cos^4\theta(z)$] *z*-magnetizations after the spatial encoding are shown in Figure S4. In the yeast experiments, the width of the spatial encoding region was 300 μm, while the flat, maximum region of the encoded magnetization $\sin^4\theta(z)$ is only about 100 μm. Close to the edges of the encoding region, non-encoded magnetization contributes to the signal significantly. Narrower spatial encoding to still narrower spatial encoding region would reduce the effect of non-encoded magnetization in UF-DEXSY experiments. On the other hand, narrower region would increase mixing between encoding layers (see below).

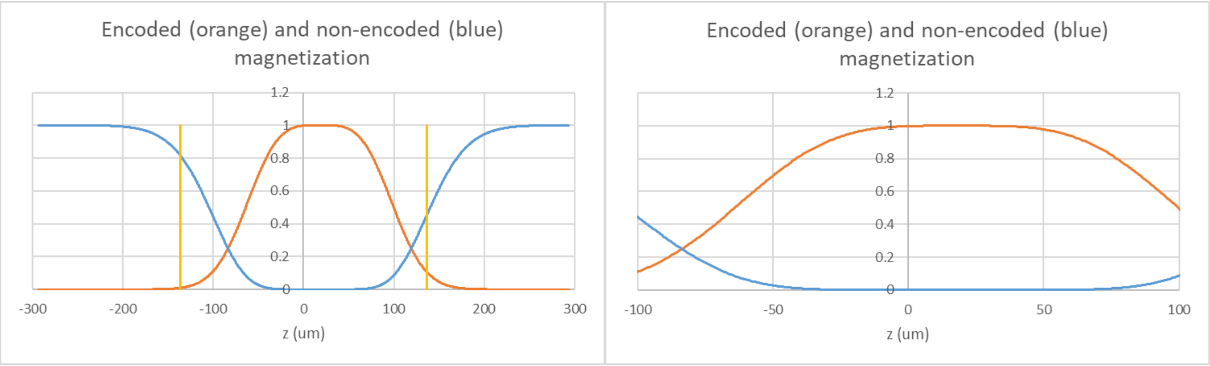

Figure S4. Left: Encoded (orange) and non-encoded (blue) magnetization after the spatial encoding, stored along the *z*-direction for the mixing period. The former is proportional to $\sin^4\theta(z)$, the latter to $\cos^4\theta(z)$. Yellow, vertical lines show the encoding region, when the chirp bandwidth is 93 kHz. Right: zoom in to the flat region (width about 100 μm).

According to the hyperpolarized CPMG reference measurements, the yeast sample includes two components with different diffusion coefficients:

1. Extracellular component with $D_1 \approx 3.2\cdot10^{-9}$ m²/s and $E_1 \approx 0.8$
2. Intracellular component with $D_2 \approx 1.0\cdot10^{-9}$ m²/s and $E_2 \approx 0.2$

Note that the values given above are approximative for illustration of the theoretical background of the UF DEXSY analysis; more accurate experimentally determined values are reported in the main article.

Ideally the longitudinal magnetization profile after the spatial encoding is

$$E_{\mathrm{e}}(z) = E_1 \exp[-D_1 b(z)] + E_2 exp[-D_2 b(z)], \quad (3)$$

where

$$b(z) = \left[\gamma G t_{\mathrm{C}} \left(1 - \frac{\gamma G z}{\pi \Delta \nu}\right)\right]^2 \Delta. \quad (4)$$

Here, γ is the gyromagnetic ratio, G is the gradient strength, t_{C} is the chirp pulse length, $\Delta\nu$ is the chirp pulse bandwidth, and Δ is the diffusion delay. The ideal magnetization profile for the yeast sample is plotted in Figure S5.

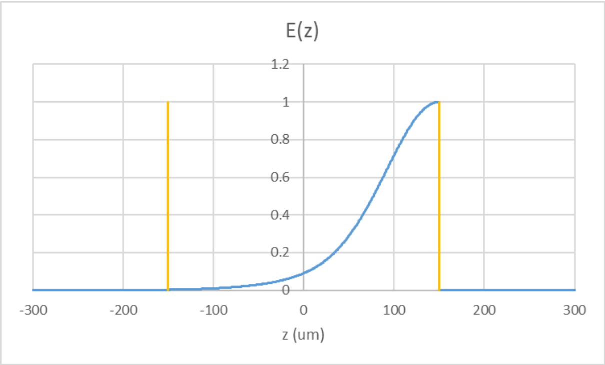

Figure S5. Ideal magnetization profile of the yeast sample after the spatial encoding. Parameters used in the plot: $D_1 = 3.2 \cdot 10^{-9}$ m^2/s, $E_1 = 0.8$, $D_2 = 1.0 \cdot 10^{-9}$ m^2/s, $E_2 = 0.2$, $\Delta = 4$ ms, $\delta = 0.6$ ms, $G = 7.28$ T/m. Yellow, vertical lines show the encoding region, when the chirp bandwidth is 93 kHz.

In practice, the encoded longitudinal is weighted by the factor of $\sin^4\theta(z)$ (orange line in Figure S4) due to B_1 inhomogeneity. Furthermore, there is non-encoded longitudinal magnetization, which is proportional to $\cos^4\theta(z)$ (blue line in Figure S4). Therefore, the total z-magnetization profile after the spatial encoding is

$$E_\mathrm{t}(z) = \sin^4\theta(z)E_\mathrm{e}(z) + \cos^4\theta(z). \tag{5}$$

This profile is illustrated in Figure S6. The encoded magnetization dominates at the center, but its maximum value is only about 0.4 due to B_1 weighting. The amount of the non-encoded magnetization is significant around $|z| \geq 100$ μm. Due to the non-encoded magnetization, the magnetization profile starts to increase at $z < -50$ μm, *i.e.*, clearly within the encoding region (observed also in the UF DEXSY yeast experiments, see Figure 2). Note that Eq. 5 and Figure S6 do not take into account the effects of artefacts around the edges of the spatial encoding region due to imperfect performance of the chirp pulse; this significantly decreases the amount of encoded magnetization around those regions. This is taken into account in the simulations described in the next section.

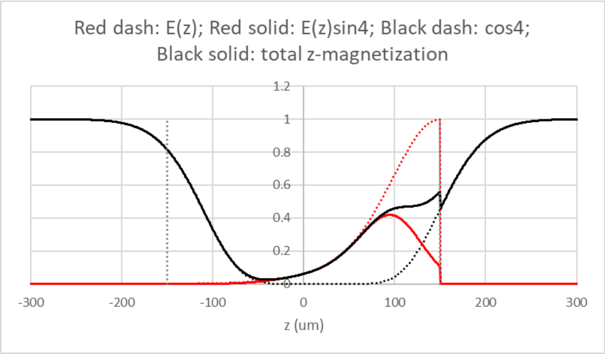

Figure S6. Black solid line: the total z-magnetization profile after the spatial encoding. Black dotted line: non-encoded magnetization, $cos^4\theta(z)$. Red dotted line: ideal encoded magnetization E_e (Eq. 3). Red solid line: B_1 weighted [$\sin^4\theta(z)$] encoded magnetization. Gray, vertical dotted lines show the spatial encoding region when the chirp bandwidth is 93 kHz.

During the mixing time τ_M, spins move from their initial position z_0 to a new position z due to molecular diffusion. Self-diffusion is characterized by the Gaussian propagator

$$P(z_0|z, \tau_M) = \frac{1}{\sqrt{4\pi Dt}} e^{-(z-z_0)^2/4D\tau_M}. \tag{6}$$

Here, D is the self-diffusion coefficient. The diffusion propagators corresponding to the three mixing time used in the yeast experiments (τ_M = 10, 30 and 100 ms) are shown in Figure S7. The diffusion coefficient of the dominant component ($D_1 = 3.2 \cdot 10^{-9}$ m^2/s) was used in the calculations of the propagators. The propagator of the shortest mixing time is narrow, with full width at half maximum (FWHM) of about 19 μm. The longest mixing time propagator is significantly wider: its FWHM is about 60 μm and full width at tenth maximum is about 110 μm. As the width of the spatial encoding region is 300 μm (and the region without non-encoded magnetization is even narrower, about 150 μm), significant mixing between the encoding layers as well as between encoded and non-encoded magnetization takes place during the longest mixing time.

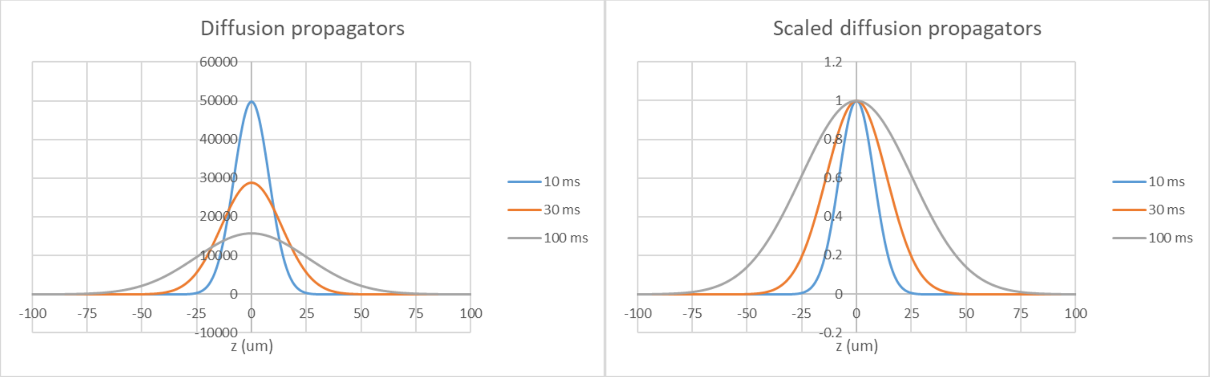

Figure S7. Left: Diffusion propagators (Eq. 6) for mixing times of τ_M = 10, 30 and 100 ms ($z_0 = 0$, $D = 3.2 \cdot 10^{-9}$ m^2/s). Right: The same propagators with maximum normalized to be 1. Root-mean-square-displacements corresponding to the three mixing times: 8.0, 14 and 25 μm.

Due to diffusional mixing, the magnetization profile after the mixing time τ_M is

$$E_d(z) = \int E_t(z_0) \int P(z_0|z, \tau_M)\, dz dz_0. \tag{7}$$

Diffusion mixed (Eq. 7) and non-mixed (Eq. 5) total z-magnetization profiles are compared in Figure S8 (mixing time: 100 ms). Due to the diffusion mixing, the slope of the curve in the encoded region is smaller, leading to lower apparent diffusion coefficient, if the diffusion mixing is not taken into account. Figures S9 and S10 show the same curves for mixing times of 10 and 30 ms; the effect of diffusional mixing is much smaller in these curves.

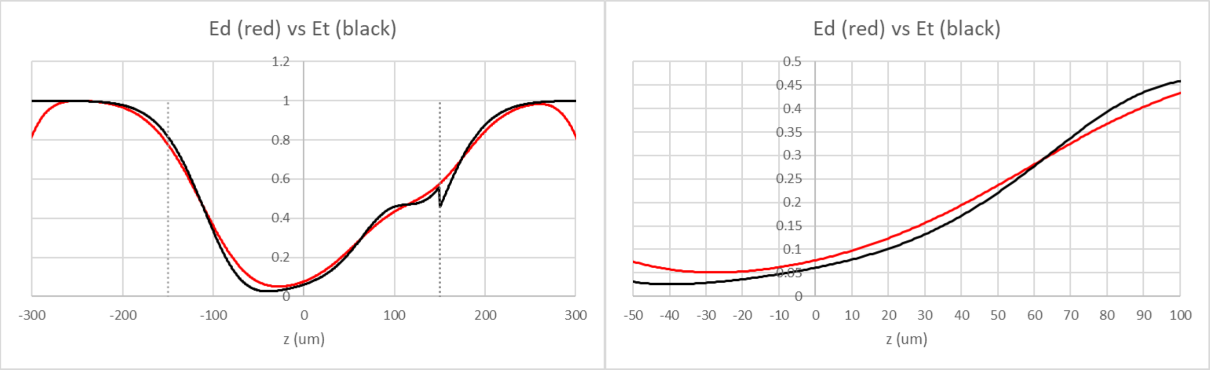

Figure S8. Left: Comparison of diffusion mixed (E_d, Eq. 7, red) and non-mixed (E_t, Eq. 5, black) total z-magnetization profiles after the spatial encoding. Gray vertical dotted lines show the spatial encoding region when the chirp bandwidth is 93 kHz. Right: zoom in the encoding region. **Mixing time: 100 ms.**

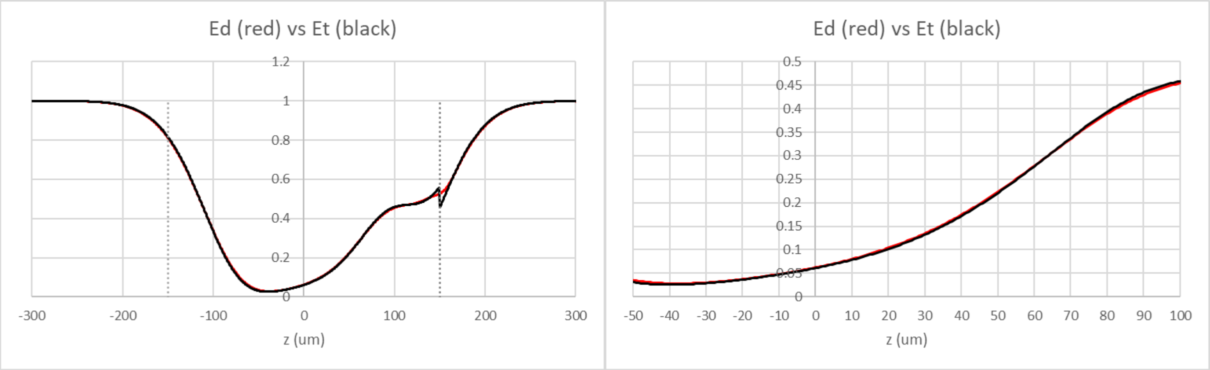

Figure S9. Like Figure S8, but **mixing time 10 ms**.

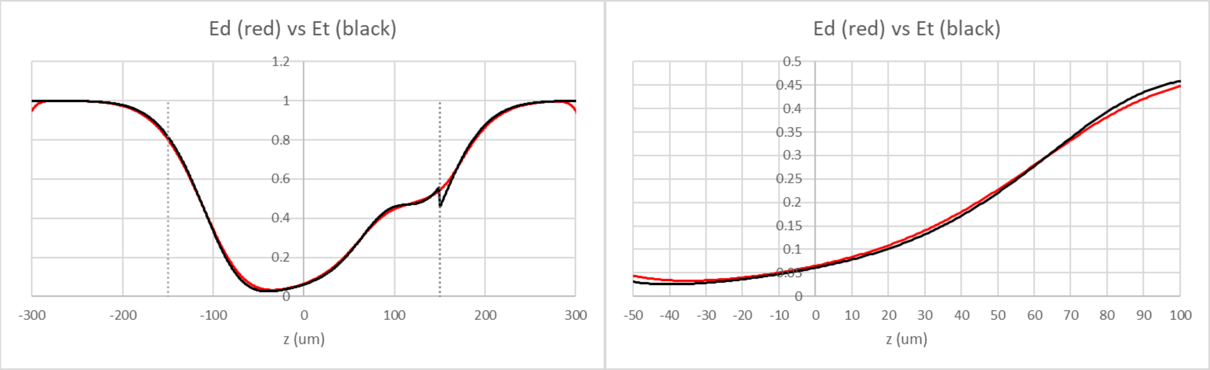

Figure S10. Like Fig. 8, but **mixing time 30 ms**.

After the mixing time, the diffusion mixed *z*-magnetization E_d is detected within the CPMG loop. The Fourier transform of the first echo is (approximately) E_d weighted by the coil excitation-detection profile, and after the coil excitation-detection correction, the image is (approximately) equivalent to E_d. Qualitatively, the experimental results shown in Figure 2 are in agreement with the theoretical curves shown Figures S8-S10:

1. The slope in the center of the encoding region decreases with increasing mixing time due to diffusional mixing.
2. The non-encoded magnetization contributes significantly to the magnetization profiles close to the edges of the encoding region, significantly narrowing the region, in which the spatial encoding is clearly visible.

More precise simulations of the UF DEXSY data, taking also into account of the chirp imperfections around the edges of the spatial encoding region as well as the intra- and extracellular exchange of hyperpolarized water during the mixing time are described in the next section.

UF DEXSY data simulations

The analysis of the UF DEXSY data required simulation of various effects described in the article and previous section. The simulated UF DEXSY data was fitted with the experimental data as illustrated in Figure S11.

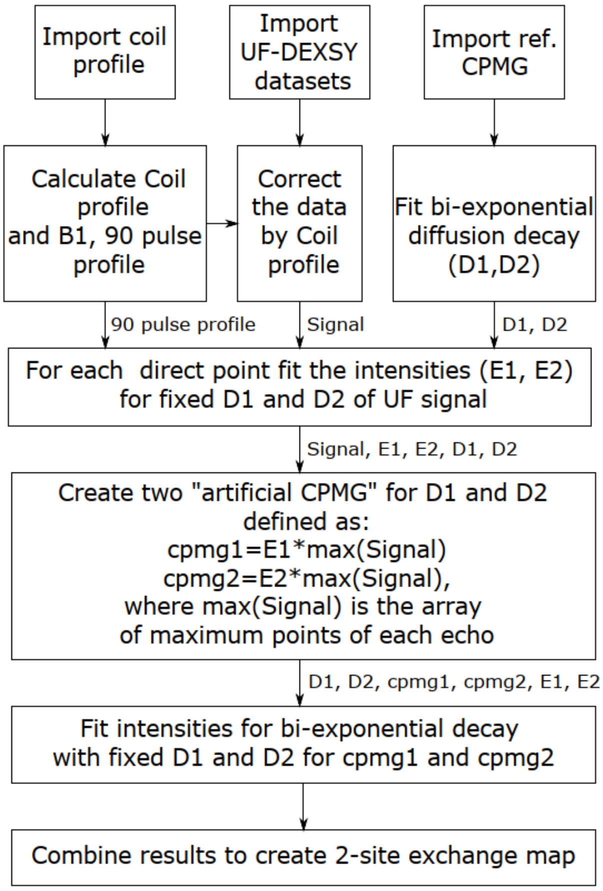

Figure S11. General scheme of fitting procedure.

An 1D MR image of a water sample (Figure 2d) was used determine the B_1 profile of the coil (see Eq. 2). This allowed the determination of the spatial dependence of true flip angle of the $\pi/2$ pulses and its effect on the encoded and non-encoded magnetization. For determining the effect of the non-ideal behavior of the chirp pulses in the beginning and at the end of sweep, we measured a coil sensitivity profile corrected magnetization profile of the water sample after the chirp sweep with a pulse sequence including a π chirp pulse followed by a hard $\pi/2$ read pulse. The observed magnetization profile could be modelled satisfactorily by the blue dashed curve shown in Figure S12, which is a sum of a constant factor and two negative gaussian functions. The zero

points of the function correspond to π/2 chirp pulse angle, while the maximum value in the center corresponds to full π chirp inversion and the maxima close to edges correspond to 0 chirp pulse angle. Hence, we were able to determine the spatially dependent chirp pulse angle, $E_{CHIRP}(z)$, shown by red line in Figure S12 (1 corresponds to flip angle of π).

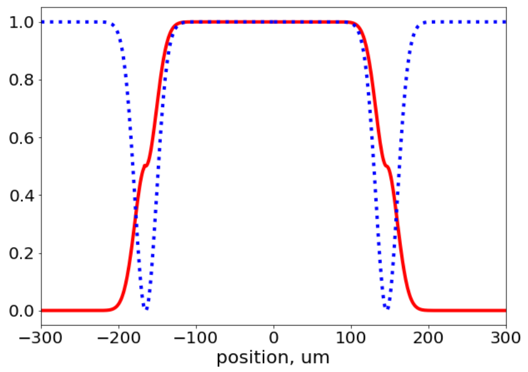

Figure S12. The chirp π pulse profile modelling. Blue dotted line: model of magnetization profile in the experiment including a π chirp pulse followed by a hard π/2 read pulse. Red-line: spatially dependent flip angle of the chirp π pulse, $E_{\mathrm{CHIRP}}(z)$, Here, 1 corresponds to flip angle of π.

The magnetization profile in the beginning of the mixing time τ_{M} is:

$$E_{\mathrm{t}}(z) = \sin^4\theta(z)E_{\mathrm{e}}(z) \cdot E_{\mathrm{CHIRP}}(z)^2 + \cos^4\theta(z) \cdot (2 \cdot E_{\mathrm{CHIRP}}(z) - 1)^2 \quad (8)$$

Eq. 8 is otherwise equivalent to Eq. 5, but there are additional factors taking into account the effect of the chirp pulse imperfections. The effect of mixing of the encoded and unencoded magnetization during the mixing time was taken into account as described by Eq. 7. To reduce the number of variables, the diffusion coefficients of the intra- and extracellular water components were fixed to be equal to the values determined by reference CPMG based diffusion experiments.

All calculations were performed in Python 3.8 using numpy scipy and matplotlib libraries. The code used in the procedure is submitted to Zenodo.org repository and is accessible at: http://dx.doi.org/10.5281/zenodo.5560815

References

1. E. Parkkinen, E. Oura, H. Suomalainen, *J. Inst. Brew*. **82**, 283-285 (1976).

2. G. J. Smits, J. C. Kapteyn, H. van den Ende, F. M. Klis, *Curr. Opin. Microbiol*. **2**, 348-352 (1999).

3. S. Hohmann, *Microbiol. Mol. Biol. Rev*. **66**, 300–372 (2002).

4. K. W. Lipsø, S. Bowen, O. Rybalko, J. H. Ardenkjær-Larsen, *J. Magn. Reson.* **274**, 65-72 (2017).

5. J. H. Ardenkjaer-Larsen, S. Bowen, J. R. Petersen, O. Rybalko, M. S. Vinding, M. Ullisch, N. C. Nielsen, *Magn. Reason. Med.* **81**, 2184-2194 (2018).

6. D. I. Hoult, R. E. Richards, *J. Magn. Reson.* **24**, 71-85 (1976).
